# L-shaped association of selenium in blood metal mixtures with the incidence of liver fibrosis/cirrhosis: NHANES 2017–2020

**DOI:** 10.1097/JS9.0000000000003102

**Published:** 2025-07-23

**Authors:** Lan Wang, Dunfeng Du, Xiaoqing Ying, Yukun Tang, Shangxin Dong, Hai Shang, Jipin Jiang, Bo Yang

**Affiliations:** a Tongji Hospital, Tongji Medical College, Huazhong University of Science and Technology, Wuhan, China; b Reproductive Medicine Center, Tongji Hospital, Tongji Medical College, Huazhong University of Science and Technology, Wuhan, China; c Institute of Organ Transplantation, Tongji Hospital, Tongji Medical College, Huazhong University of Science and Technology; Key Laboratory of Organ Transplantation, Ministry of Education; NHC Key Laboratory of Organ Transplantation; Key Laboratory of Organ Transplantation, Chinese Academy of Medical Sciences; Organ Transplantation Clinical Medical Research Center of Hubei Province, Wuhan, China

**Keywords:** chronic liver disease, mixed metals, NHANES, selenium

## Abstract

**Introduction:**

Trace elements in the environment are considered significant risk factors for chronic liver disease. However, the impact of blood metal levels on the incidence of liver fibrosis or cirrhosis has not been fully assessed. Our study investigated the correlation between metal mixture exposure and liver fibrosis/cirrhosis as well as the mediating effect between non-alcoholic fatty liver disease (NAFLD) and liver fibrosis/cirrhosis.

**Methods:**

We conducted a cross-sectional study using the National Health and Nutrition Examination Survey database from 2017 to 2020. Transient elastography was used to identify NAFLD and liver fibrosis or cirrhosis. Various statistical models have been applied to evaluate the relationship between blood metal mixtures and liver fibrosis/cirrhosis. The role of metal mixtures in NAFLD-associated liver fibrosis/cirrhosis was explored using a mediation analysis.

**Results:**

In the fully adjusted logistic models, selenium was negatively correlated with liver fibrosis [odds ratio (OR) = 0.219, *P* = 0.039) and cirrhosis (OR = 0.007, *P* = 0.003). In the weighted quantile sum and quantile-based g-computation models, selenium showed the highest contribution to liver fibrosis (0.544 and 0.578) and cirrhosis (0.538 and 0.630, respectively). Bayesian kernel machine regression and restricted cubic splines models indicated an L-shaped association between selenium and liver fibrosis/cirrhosis. Selenium negatively mediated the relationship between NAFLD and liver fibrosis/cirrhosis according to the mediation analysis (−3.4% and −13.3%).

**Conclusions:**

Selenium shows an L-shaped relationship with liver fibrosis and cirrhosis and plays a negative mediating role in the association between NAFLD and liver fibrosis/cirrhosis. Blood selenium is a promising biomarker for chronic liver diseases, and supplemental selenium intake might contribute to the prevention and management of liver fibrosis/cirrhosis.

## Introduction

Liver fibrosis and cirrhosis represent significant drivers of perioperative morbidity and mortality among patients undergoing liver surgery procedures such as hepatic resection or transplantation^[1]^. Liver fibrosis and cirrhosis are the terminal stages of most chronic liver diseases^[2]^ and can lead to adverse outcomes such as hepatocellular carcinoma, hepatic decompensation, and hepatic encephalopathy^[3,4]^. Globally, cirrhosis has a prevalence of nearly 1.4%^[5]^ and is associated with 2.4% global mortality^[6]^. Precise assessment of fibrosis/cirrhosis severity plays a pivotal role in guiding surgical decisions and improving outcomes.

Emerging evidence has linked metal exposure to hepatic toxicity^[7]^. Single-metal exposures, such as mercury, lead, and cadmium, are associated with impaired liver function impairment^[8–10]^. Simultaneous exposure to multiple environmental chemicals may lead to interactions between co-exposure and confounding research on their effects^[11,12]^. For example, selenium antagonizes cadmium across diverse physiological and pathological conditions^[13,14]^. Thus, investigating the association between mixed metal exposure, hepatic fibrosis, and cirrhosis is necessary.

The association between blood metal mixture content and the risk of liver fibrosis or cirrhosis, as well as their dose–response relationships, remains unclear. This study used multivariate logistic models, restricted cubic splines (RCS) models, weighted quantile sum (WQS) regression, quantile-based g-computation (qgcomp), and Bayesian kernel machine regression (BKMR) to investigate the association between metal co-exposure and liver fibrosis/cirrhosis risk. Additionally, we explored whether mixed metals mediated the association between non-alcoholic fatty liver disease (NAFLD) and liver fibrosis/cirrhosis, as NAFLD is a significant cause of liver fibrosis or cirrhosis^[15]^.

## Methods

### Study population

**Figure 1.**
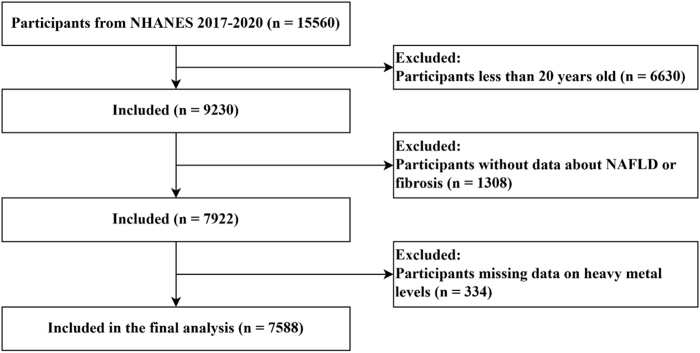
Selection flowcharts.

We included all participants (*n* = 15 560) using the National Health and Nutrition Examination Survey (NHANES) database from 2017 to 2020 (http://www.cdc.gov/nchs/nhanes.htm). Participants aged under 20 years (*n* = 6630), those missing vibration-controlled transient elastography (VCTE) data (*n* = 1308), and those missing blood metal information (*n* = 334) were excluded. In total, 7588 individuals were included in the final analysis (Fig. 1).

### Definitions of NAFLD, liver fibrosis, and cirrhosis

These definitions were based on previous studies^[16,17]^. All participants aged ≥12 years underwent transient elastography. Participants were excluded if they were unable to lie on the examination bed, were pregnant, had implanted electronic medical devices, wore bandages or lesions near the right upper abdomen, or refused the examination. NAFLD was defined as a controlled attenuation parameter of ≥263 dB/m^[18]^. Liver stiffness measurement (LSM) values of ≥8 kPa and ≥13.1 kPa indicated liver fibrosis and cirrhosis, respectively, in the absence of other liver disease causes^[19,20]^.

### Blood heavy metals measurement

Blood samples were collected from all participants at mobile examination centers. The samples were processed, stored, and transported to the National Center for Environmental Health for testing and analysis. Lead, cadmium, mercury, selenium, and manganese levels in whole blood were measured using inductively coupled plasma mass spectrometry, with concentrations reported in μg/L. Values below the limit of detection were omitted, and rigorous quality control protocols were implemented to guarantee the precision and dependability of the measurements.

**HIGHLIGHTS**

- In the context of blood mixed metal elements, the level of blood selenium plays a dominant role and is negatively correlated with hepatic fibrosis/cirrhosis.
- The association between selenium and liver fibrosis/cirrhosis exhibits an L-shaped pattern.
- Selenium negatively mediates the association between NAFLD and liver fibrosis/cirrhosis.

### Covariates

Demographic characteristics were collected via personal and household questionnaires, including age, sex, race, poverty-income ratio (PIR), education, marital status, insurance, smoking status, alcohol consumption, physical activity, diabetes, and hypertension. The race categories included Mexican American, other Hispanics, non-Hispanic white, non-Hispanic black, and others. Education was divided into three levels: high school, high school or equivalent, and higher than high school. The PIR was categorized as ≤1.0 (poverty) or >1.0 (non-poverty). Marital status was categorized as married/living with a partner or widowed/divorced/separated/never married.

### Statistical analysis

Chi-squared and *t*-tests were used to assess the demographic characteristics related to NAFLD status. The blood heavy metal concentrations underwent log10 transformation and were categorized into quartiles. Pearson’s correlation coefficients were calculated to assess the associations among metal levels. Univariate and multivariate logistic regression analyses were used to estimate the association of blood metal levels with NAFLD, liver fibrosis, and cirrhosis. Model 1 was adjusted for age, sex, and body mass index (BMI), while Model 2 was additionally adjusted for race, marital status, education, PIR, insurance, alcohol consumption, smoking status, physical activity, hypertension, and diabetes. Missing covariate data were addressed through imputation via the multiple imputation by chained equations method. Trend tests across the groups were performed using integer values. RCS and segmented logistic regression were used to examine the nonlinear associations between single exposure and liver fibrosis/cirrhosis.

WQS regression was used to explore the overall impact of the metal mixtures on liver fibrosis and cirrhosis. The WQS index, calculated as the weighted sum of individual metal concentrations, represents the mixed exposure level, with non-negligible weights identifying the key components^[21]^. Bootstrapping with 10 000 iterations constructed the WQS index, and weights determined the relative contribution of each metal to disease prevalence.

qgcomp integrates the simplicity of WQS with the adaptability of g-computation, obviating the need for homogeneity assumptions or constraints on the linearity/additivity of exposure. It provides consistent estimates of exposure mixtures in environments where WQS might be biased^[22]^. qgcomp yielded equivalent estimates of WQS in large samples when assumptions were made^[23]^.

BKMR with a Gaussian kernel function was used to assess the combined effects of blood metal mixtures and dose–response associations between individual metals and liver fibrosis/cirrhosis, accounting for potential nonlinear and non-additive relationships^[24]^.

A mediation analysis was performed to investigate the mediating effect of metals on the association between NAFLD and liver fibrosis/cirrhosis, adjusting for all covariates. All analyses were performed in R (version 4.3.2), considering the complex sampling design of the NHANES, with a two-sided significance *P* level of 0.05.

This study was conducted following the guideline of strengthening the reporting of cohort, cross-sectional, and case–control studies in surgery (STROCSS 2025)^[25]^.

## Results

### Descriptive statistics

The demographic characteristics of 7588 individuals from NHANES 2017–2020 are presented in Table 1. In the study population, the mean age was 48.21 ± 0.57 years, the average BMI was 29.72 ± 0.18 kg/m^2^, and female participants comprised 50.88% of the participants. Of these, 3874 (51.05%) were classified as NAFLD, 868 (11.44%) were diagnosed with liver fibrosis, and 267 (3.52%) were diagnosed with cirrhosis. NAFLD participants had a higher prevalence of liver fibrosis (17.63% vs. 4.98%, *P* < 0.001) and cirrhosis (5.50% vs. 1.45%, *P* < 0.001) than non-NAFLD participants. Supplemental Digital Content Fig. S1, available at: http://links.lww.com/JS9/E769, shows the Spearman correlation coefficients for the heavy metal concentrations. Rho between lead and manganese is 0.17 (*P* < 0.05), and rho between mercury and selenium is 0.17 (*P* < 0.05).

**Table 1:** Baseline characteristics based on NAFLD present

Variables	Total	NAFLD	Non-NAFLD	*P*
Age	48.21 (0.57)	51.00 (0.58)	45.43 (0.66)	<0.001
BMI	29.72 (0.18)	33.28 (0.22)	26.16 (0.17)	<0.001
Gender				<0.001
Male	3727 (49.12)	2059 (53.15)	1668 (44.91)	
Female	3861 (50.88)	1815 (46.85)	2046 (55.09)	
Race				<0.001
Mexican American	918 (12.10)	602 (15.54)	316 (8.51)	
Non-Hispanic Black	805 (10.61)	421 (10.87)	384 (10.34)	
Non-Hispanic White	2630 (34.66)	1395 (36.01)	1235 (33.25)	
Other Hispanic	1956 (25.78)	842 (21.73)	1114 (29.99)	
Other race	1279 (16.86)	614 (15.85)	665 (17.91)	
Education				0.008
Under high school	583 (7.68)	342 (8.83)	241 (6.49)	
High school or equivalent	2664 (35.11)	1363 (35.18)	1301 (35.03)	
Above high school	4341 (57.21)	2169 (55.99)	2172 (58.48)	
Marital				<0.001
Married/living with partner	4405 (58.05)	2393 (61.77)	2012 (54.17)	
Widowed/divorced/separated/never married	3183 (41.95)	1481 (38.23)	1702 (45.83)	
PIR				0.932
Poverty	1466 (19.32)	729 (18.82)	737 (19.84)	
Non-poverty	6122 (80.68)	3145 (81.18)	2977 (80.16)	
Insurance				0.056
Yes	6342 (83.58)	3274 (84.51)	3068 (82.61)	
No	1246 (16.42)	600 (15.49)	646 (17.39)	
Smoke				0.109
Yes	3181 (41.92)	1682 (43.42)	1499 (40.36)	
No	4407 (58.08)	2192 (56.58)	2215 (59.64)	
Alcohol				0.002
Yes	1126 (14.84)	622 (16.06)	504 (13.57)	
No	6462 (85.16)	3252 (83.94)	3210 (86.43)	
Hypertension				<0.001
Yes	2907 (38.31)	1801 (46.49)	1106 (29.78)	
No	4681 (61.69)	2073 (53.51)	2608 (70.22)	
Diabetes				<0.001
Yes	1369 (18.04)	979 (25.27)	390 (10.50)	
No	6219 (81.96)	2895 (74.73)	3324 (89.50)	
Physical activity				0.710
High	3370 (44.41)	1753 (45.25)	1617 (43.54)	
Low	4218 (55.59)	2121 (54.75)	2097 (56.46)	
Cadmium, µg/dL	0.43 (0.01)	0.40 (0.01)	0.46 (0.02)	0.003
Manganese, µg/dL	9.65 (0.06)	9.94 (0.11)	9.37 (0.07)	<0.001
Lead, µg/dL	1.08 (0.02)	1.10 (0.03)	1.05 (0.03)	0.279
Selenium, µg/dL	188.45 (0.90)	190.04 (1.02)	186.87 (1.01)	<0.001
Mercury, µg/dL	1.34 (0.05)	1.30 (0.07)	1.37 (0.07)	0.421
LDL, mg/dL	90.75 (1.28)	99.94 (1.31)	81.58 (1.51)	<0.001
HDL, mg/dL	53.75 (0.44)	49.20 (0.49)	58.29 (0.52)	0.000
TG, mg/dL	223.03 (5.21)	214.97 (5.90)	231.08 (7.42)	0.281
TCHO, mg/dL	187.19 (1.17)	190.81 (1.53)	183.57 (1.14)	<0.001
ALT, U/L	22.77 (0.32)	26.54 (0.50)	19.02 (0.35)	<0.001
AST, U/L	21.85 (0.23)	23.00 (0.31)	20.70 (0.31)	<0.001
ALP, IU/L	75.14 (0.51)	78.79 (0.63)	71.51 (0.87)	<0.001
GGT, IU/L	29.40 (0.57)	35.23 (0.76)	23.58 (0.78)	<0.001
Fibrosis				<0.001
Yes	868 (11.44)	683 (17.63)	185 (4.98)	
No	6720 (88.56)	3191 (82.37)	3529 (95.02)	
Cirrhosis				<0.001
Yes	267 (3.52)	213 (5.50)	54 (1.45)	
No	7321 (96.48)	3661 (94.50)	3660 (98.55)	

Mean ± SE for continuous variables, *n* (%) for categorical variables.

PIR, poverty to income ratio; NAFLD, non-alcoholic fatty liver disease; BMI, body mass index; LDL, low-density lipoprotein; HDL, high-density lipoprotein; TG, triglycerides; TCHO, total cholesterol; ALT, alanine aminotransferase; AST, aspartate aminotransferase; ALP, alkaline phosphatase; GGT, gamma-glutamyl transferase.

### Logistic and RCS models for single metal analysis

Logistic models were used to fit liver fibrosis and cirrhosis. Tables 2 and 3 demonstrate that in fully adjusted models, blood selenium as a continuous variable was significantly negatively associated with liver fibrosis [odds ratio (OR) = 0.219, 95% CI: 0.053–0.910, *P* = 0.039] and cirrhosis (OR = 0.007, 95% CI: 0.000–0.111, *P* = 0.003). Blood mercury was negatively associated with liver fibrosis (OR = 0.452, 95% CI: 0.278–0.734, *P* = 0.004), but not with cirrhosis (OR = 0.674, 95% CI: 0.194–2.348, *P* = 0.498). Supplemental Digital Content Figure S2, available at: http://links.lww.com/JS9/E769, shows the Spearman correlation between the serum element levels and chronic liver diseases.

**Table 2:** Logistic models for liver fibrosis

Variables	Crude model		Model 1		Model 2	
	OR (95% CI)	*P*	OR (95% CI)	*P*	OR (95% CI)	*P*
Cadmium	0.524 (0.286–0.957)	0.037	1.682 (0.727–3.891)	0.211	1.420 (0.519–3.884)	0.455
Q1	Ref		Ref		Ref	
Q2	1.176 (0.851–1.625)	0.311	1.194 (0.816–1.747)	0.336	1.168 (0.774–1.762)	0.421
Q3	1.153 (0.730–1.822)	0.525	1.034 (0.608–1.761)	0.894	0.972 (0.554–1.703)	0.912
Q4	1.314 (1.049–1.647)	0.020	0.940 (0.708–1.250)	0.651	0.904 (0.658–1.241)	0.494
	*P* for trend	0.311	*P* for trend	0.336	*P* for trend	0.421
Manganese	2.759 (1.401–5.431)	0.005	2.549 (0.999–6.499)	0.050	2.445 (0.930–6.427)	0.066
Q1	Ref		Ref		Ref	
Q2	0.983 (0.662–1.461)	0.931	1.079 (0.706–1.648)	0.707	1.060 (0.681–1.650)	0.776
Q3	0.956 (0.644–1.418)	0.814	1.037 (0.722–1.490)	0.833	1.013 (0.691–1.487)	0.941
Q4	0.769 (0.596–0.992)	0.044	0.848 (0.606–1.187)	0.312	0.810 (0.564–1.162)	0.222
	*P* for trend	0.931	*P* for trend	0.707	*P* for trend	0.776
Lead	1.250 (0.689–2.268)	0.447	1.638 (0.695–3.863)	0.245	1.988 (0.766–5.157)	0.139
Q1	Ref		Ref		Ref	
Q2	0.977 (0.695–1.373)	0.889	0.978 (0.654–1.461)	0.905	0.899 (0.596–1.356)	0.577
Q3	0.976 (0.737–1.293)	0.858	1.012 (0.641–1.597)	0.956	0.918 (0.570–1.477)	0.697
Q4	1.002 (0.788–1.274)	0.986	0.925 (0.650–1.317)	0.645	0.782 (0.533–1.148)	0.185
	*P* for trend	0.889	*P* for trend	0.905	*P* for trend	0.577
Selenium	0.266 (0.055–1.295)	0.097	0.198 (0.050–0.786)	0.023	0.219 (0.053–0.910)	0.039
Q1	Ref		Ref		Ref	
Q2	0.927 (0.701–1.227)	0.580	0.913 (0.680–1.224)	0.515	0.903 (0.672–1.212)	0.456
Q3	1.018 (0.724–1.431)	0.913	1.038 (0.735–1.466)	0.819	1.032 (0.744–1.432)	0.834
Q4	1.067 (0.768–1.483)	0.687	1.170 (0.847–1.614)	0.315	1.173 (0.840–1.637)	0.312
	*P* for trend	0.580	*P* for trend	0.515	*P* for trend	0.456
Mercury	0.353 (0.233–0.534)	<0.001	0.363 (0.219–0.603)	<0.001	0.452 (0.278–0.734)	0.004
Q1	Ref		Ref		Ref	
Q2	1.036 (0.730–1.472)	0.834	1.131 (0.790–1.620)	0.474	1.113 (0.754–1.644)	0.554
Q3	1.028 (0.722–1.464)	0.874	1.085 (0.744–1.582)	0.650	1.040 (0.677–1.599)	0.843
Q4	1.624 (1.243–2.122)	0.001	1.623 (1.189–2.216)	0.005	1.570 (1.130–2.180)	0.012
	*P* for trend	0.834	*P* for trend	0.474	*P* for trend	0.554

OR, odds ratio; CI, confidence interval; Ref, reference. Model 1 adjusted for age, gender, and BMI; Model 2 additionally adjusted for race, marital status, education, poverty to income ratio, insurance, smoking, alcohol, physical activity, hypertension, and diabetes.

**Table 3:** Logistic models for cirrhosis

Variables	Crude model		Model 1		Model 2	
	OR (95% CI)	*P*	OR (95% CI)	*P*	OR (95% CI)	*P*
Cadmium	0.501 (0.145–1.732)	0.261	2.309 (0.716–7.445)	0.152	1.894 (0.545–6.583)	0.280
Q1	Ref		Ref		Ref	
Q2	1.013 (0.647–1.587)	0.952	0.974 (0.593–1.598)	0.910	0.950 (0.566–1.593)	0.828
Q3	1.189 (0.696–2.032)	0.509	0.968 (0.518–1.810)	0.914	0.926 (0.474–1.809)	0.805
Q4	1.194 (0.785–1.816)	0.390	0.715 (0.441–1.159)	0.159	0.706 (0.425–1.172)	0.157
	*P* for trend	0.952	*P* for trend	0.910	*P* for trend	0.828
Manganese	4.166 (1.064–16.308)	0.041	3.983 (0.665–23.838)	0.123	2.854 (0.433–18.813)	0.244
Q1	Ref		Ref		Ref	
Q2	1.341 (0.697–2.580)	0.363	1.602 (0.719–3.567)	0.228	1.586 (0.697–3.610)	0.240
Q3	0.854 (0.610–1.194)	0.339	0.928 (0.579–1.487)	0.739	0.947 (0.570–1.573)	0.815
Q4	0.741 (0.444–1.236)	0.238	0.880 (0.440–1.760)	0.698	0.864 (0.414–1.803)	0.668
	*P* for trend	0.363	*P* for trend	0.228	*P* for trend	0.240
Lead	1.383 (0.330–5.793)	0.644	2.777 (0.529–14.592)	0.214	3.336 (0.608–18.312)	0.146
Q1	Ref		Ref		Ref	
Q2	0.881 (0.457–1.697)	0.692	0.743 (0.341–1.619)	0.427	0.682 (0.324–1.437)	0.279
Q3	1.191 (0.596–2.379)	0.607	1.035 (0.405–2.645)	0.938	0.953 (0.377–2.408)	0.911
Q4	0.941 (0.475–1.864)	0.855	0.669 (0.273–1.637)	0.351	0.578 (0.241–1.385)	0.193
	*P* for trend	0.692	*P* for trend	0.427	*P* for trend	0.279
Selenium	0.010 (0.001–0.127)	0.001	0.007 (0.001–0.103)	0.001	0.007 (0.000–0.111)	0.003
Q1	Ref		Ref		Ref	
Q2	0.904 (0.614–1.331)	0.593	0.863 (0.569–1.308)	0.459	0.847 (0.538–1.333)	0.434
Q3	1.637 (1.043–2.567)	0.033	1.806 (1.011–3.226)	0.046	1.795 (0.996–3.238)	0.051
Q4	1.612 (1.045–2.487)	0.032	1.921 (1.226–3.009)	0.008	1.929 (1.234–3.013)	0.008
	*P* for trend	0.593	*P* for trend	0.459	*P* for trend	0.434
Mercury	0.435 (0.149–1.271)	0.122	0.518 (0.147–1.823)	0.289	0.674 (0.194–2.348)	0.498
Q1	Ref		Ref		Ref	
Q2	1.106 (0.626–1.951)	0.718	1.192 (0.640–2.217)	0.555	1.176 (0.616–2.247)	0.589
Q3	1.380 (0.727–2.619)	0.309	1.481 (0.793–2.764)	0.199	1.434 (0.740–2.780)	0.252
Q4	1.273 (0.766–2.114)	0.335	1.190 (0.638–2.218)	0.559	1.160 (0.610–2.207)	0.618

OR, odds ratio; CI, confidence interval; Ref, reference. Model 1 adjusted for age, gender, and BMI; Model 2 additionally adjusted for race, marital status, education, poverty to income ratio, insurance, smoking, alcohol, physical activity, hypertension, and diabetes.

**Figure 2.**
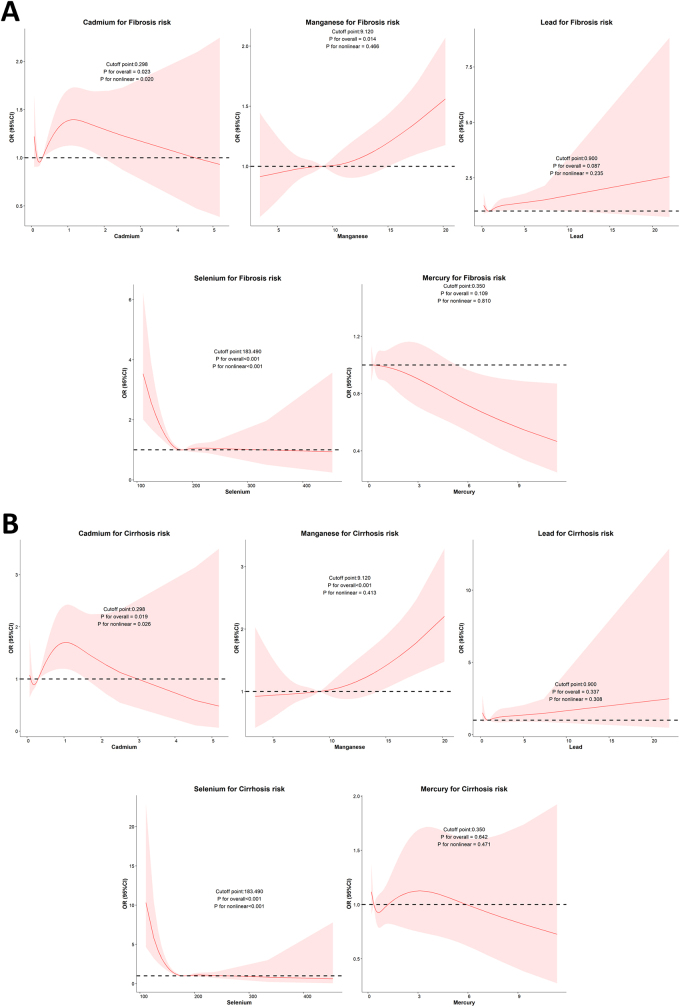
RCS analysis for every metal on (A) liver fibrosis and (B) cirrhosis.

RCS analysis (Fig. 2) and segmented logistic regression (Supplemental Digital Content, Table S1, available at: http://links.lww.com/JS9/E770) revealed L-shaped associations between selenium and liver fibrosis (*P* for nonlinearity < 0.001, inflection point = 183.49 μg/L) and cirrhosis (*P* for nonlinearity < 0.001, inflection point = 183.49 μg/L). Additionally, inverse L-shaped associations were found between cadmium levels and hepatic fibrosis (*P* for nonlinearity = 0.020) and cirrhosis (*P* for nonlinearity = 0.026).

### Associations between mixed metals and liver fibrosis/cirrhosis in WQS, qgcomp, and BKMR models

**Figure 3.**
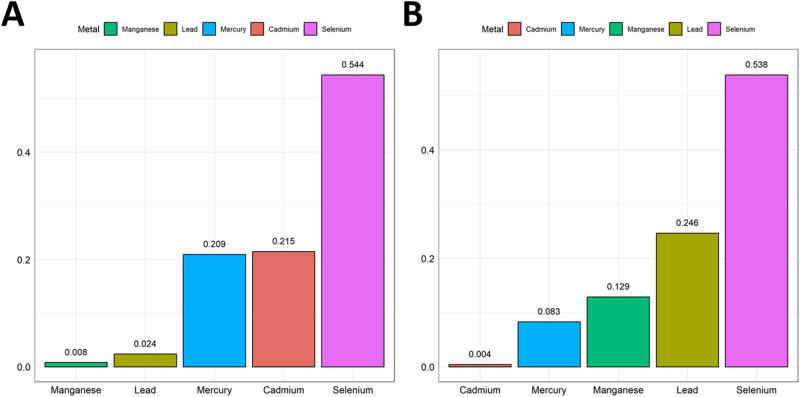
The WQS model weights of heavy metals on the prevalence of (A) liver fibrosis and (B) cirrhosis in a positive direction. This model was adjusted for all covariates.

**Figure 4.**
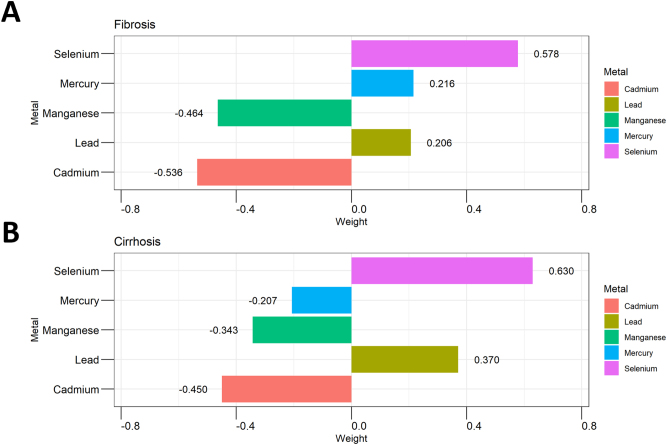
The qgcomp model weights of heavy metals on the prevalence of (A) liver fibrosis and (B) cirrhosis in a positive direction. This model was adjusted for all covariates.

In the unadjusted model (Supplemental Digital Content, Table S2, available at: http://links.lww.com/JS9/E770), the WQS index was inversely associated with liver fibrosis (OR = 0.867, 95% CI: 0.754–0.997, *P* = 0.046), but not after full adjustment (OR = 0.904, 95% CI: 0.772–1.058, *P* = 0.183). The WQS index remained significantly negatively associated with cirrhosis (OR = 0.717, 95% CI: 0.559–0.919, *P* = 0.014) in the fully adjusted models (Supplemental Digital Content, Table S2, available at: http://links.lww.com/JS9/E770). In the WQS model (Fig. 3), selenium had the highest weight among the metals (liver fibrosis, 0.544; cirrhosis, 0.538). qgcomp (Fig. 4) also highlighted the key role of selenium level (liver fibrosis, 0.578; cirrhosis, 0.630).

**Figure 5.**
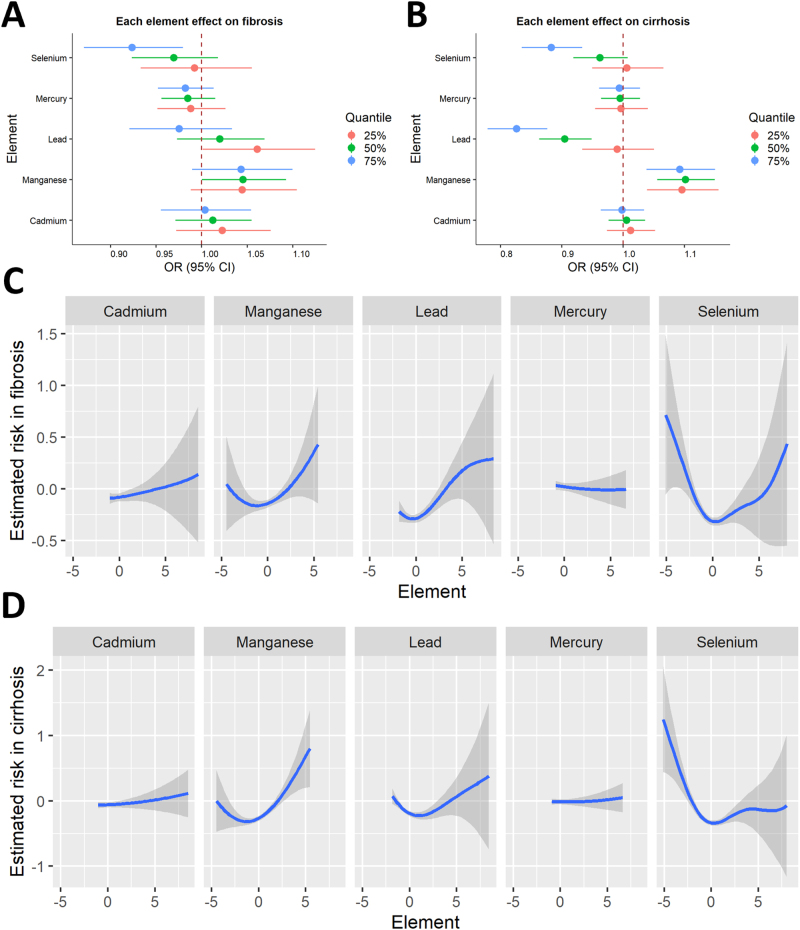
The BKMR model results of heavy metals on the prevalence of fibrosis and cirrhosis. These models were adjusted for all covariates. Single metal effects on (A) liver fibrosis and (B) cirrhosis. Nonlinear effects of each metal on (C) liver fibrosis and (D) cirrhosis.

BKMR analysis showed an unclear relationship between mixed metals and hepatic fibrosis, but a negative association with cirrhosis (Supplemental Digital Content, Fig. S3, available at: http://links.lww.com/JS9/E769). For liver fibrosis, selenium and lead had posterior inclusion probabilities (PIPs) of 1.000, whereas for cirrhosis, selenium, lead, and manganese had PIPs of 1.000 (Supplemental Digital Content, Table S3, available at: http://links.lww.com/JS9/E770). Single-metal analysis indicated that manganese was positively associated with cirrhosis risk when other metals were controlled at 25%, 50%, and 75%. When the other metals were controlled at 75%, selenium was significantly negatively associated with liver fibrosis and cirrhosis (Fig. 5A, B). Dose–response analysis showed L-shaped associations between blood selenium levels and liver fibrosis/cirrhosis, while manganese showed a J-shaped positive correlation (Fig. 5C, D).

### Mediation analysis

In the fully adjusted logistic models, manganese (OR = 2.933, 95% CI: 1.461–5.888, *P* = 0.006) and selenium (OR = 7.403, 95% CI: 1.635–33.530, *P* = 0.014) were significantly positively associated with NAFLD (Supplemental Digital Content, Table S4, available at: http://links.lww.com/JS9/E770). Among the five metals, only selenium had a significant indirect effect, mediating −3.378% of the association with liver fibrosis and −13.272% with cirrhosis (Table 4).

**Table 4:** Mediation analysis for the effects of heavy metals on liver fibrosis or cirrhosis.

Outcomes	Mediators	Indirect effect	Direct effect	Total effect	Mediation proportion (%)
Fibrosis	Mix	−0.001	0.052***	0.051***	−1.521
	Cadmium	<0.001	0.052***	0.051***	−0.456
	Manganese	0.001	0.051***	0.051***	1.341
	Lead	0.000	0.052***	0.052***	−0.120
	Selenium	−0.002**	0.053***	0.051***	−3.378
	Mercury	<0.001	0.052***	0.051***	−0.268
Cirrhosis	Mix	<0.001	0.010*	0.010*	−4.642
	Cadmium	<0.001	0.010*	0.010*	−1.403
	Manganese	0.001	0.009*	0.010*	5.489
	Lead	<0.001	0.010*	0.010*	−0.128
	Selenium	−0.001**	0.011*	0.010*	−13.272
	Mercury	<0.001	0.010*	0.010*	−0.129

Mediation model adjusted for age, gender, BMI, race, marital status, education, poverty to income ratio, insurance, smoke, alcohol, physical activity, hypertension, and diabetes. *, P < 0.05; **, P < 0.01; ***, P < 0.001.

### Subgroup analysis

**Figure 6.**
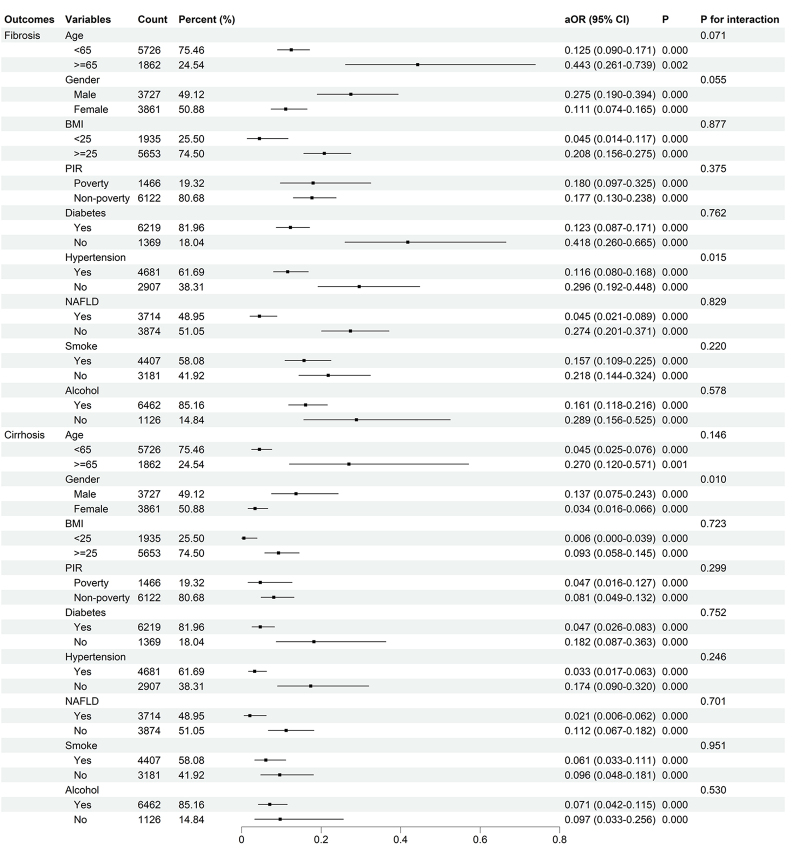
Subgroup analysis for the association between selenium and liver fibrosis/cirrhosis.

Subgroup analysis was performed to explore the effects of blood selenium levels across different subgroups (Fig. 6). Hypertensive patients were more sensitive to the protective effect of selenium against liver fibrosis (OR = 0.116, 95% CI: 0.080–0.168, *P* < 0.001) than non-hypertensive patients (OR = 0.296, 95% CI: 0.192–0.448, *P* < 0.001, *P* for interaction = 0.015). Females appeared to benefit more from serum selenium in protecting against liver fibrosis (OR = 0.111, 95% CI: 0.074–0.165, *P* < 0.001, *P* for interaction = 0.055) and cirrhosis (OR = 0.034, 95% CI: 0.016–0.066, *P* < 0.001, *P* for interaction = 0.010).

## Discussion

To our knowledge, this study is the first to systematically analyze the association between blood mixed metal exposure and the risk of liver fibrosis/cirrhosis. Across multiple statistical models, blood selenium levels were closely associated with liver fibrosis/cirrhosis risk, whereas other heavy metals were not. This study provides new insights into the relationship between environmental heavy metals and liver health. Notably, selenium has the potential to act as a non-invasive biomarker, supplementing current surgical risk assessment tools and informing perioperative management strategies.

Previous studies of single-metal exposure and liver function impairment have yielded conflicting results^[7]^. For example, Lee *et al* found significant correlations between blood mercury levels and liver function biomarkers^[8]^, whereas Choi *et al* found no significant relationship in a longitudinal study^[26]^. Similar inconsistencies exist in studies of lead and manganese^[27,28]^. The complex interactions between trace elements may explain these discrepancies. Li *et al* used mixture models to evaluate the association of blood mixed metals with liver function biomarkers and found that mixed metal exposure was positively associated with liver function markers, with different metals contributing maximally to each marker^[7]^. Significant links were only found in females in the study of the association between urine mixed metals and NAFLD^[29]^. Our study, using blood mixed metal exposure, showed that the WQS index was negatively associated with cirrhosis risk after full adjustment (OR = 0.717, 95% CI: 0.559–0.919, *P* = 0.014, *P* for trend = 0.015). Blood levels better reflect the systemic heavy metal burden than urine levels and are widely used in most studies^[30]^.

Existing research studies indicate that selenium deficiency is associated with cirrhosis^[31]^, hepatic encephalopathy^[32]^, liver cancer^[33,34]^, and poor prognosis after liver transplantation^[35]^. In healthy individuals, serum selenium is comprised of selenoproteins, selenomethionine, and other forms. Antioxidant selenoprotein glutathione peroxidase 3 protects membrane lipids from free radicals and reactive oxygen species^[36]^. Selenium can reduce inflammation and oxidative stress by enhancing the activity of glutathione peroxidase and serving as a component of anti-inflammatory selenoproteins^[37]^. Selenium deficiency decreases the expression of intracellular antioxidant enzymes, thereby promoting the progression of liver fibrosis and fatty liver^[38]^. Selenium also inhibits the fibrotic process by reducing the number of collagen-producing hepatic stellate cells and enhancing collagen degradation^[39]^, as well as by inhibiting multiple cytokines^[31,40]^. However, the preventive value of selenium in mixtures for liver fibrosis/cirrhosis has not yet been evaluated. The findings of this study confirmed the importance of selenium in protecting liver function within mixed elements. Supplementation and monitoring of selenium are expected to play crucial roles in the clinical management of chronic liver diseases. Furthermore, our study revealed an L-shaped association between selenium levels and the risk of liver fibrosis and cirrhosis. Serum selenium levels beyond a certain threshold no longer confer any benefits. Considering the toxicity of excessive selenium^[41–44]^, this threshold effect needs to be considered when reducing the risk of chronic liver diseases through selenium supplementation. It is important to note that selenium levels vary significantly among different cohorts^[45]^. Decisions regarding selenium supplementation should be made based on the different populations and baseline selenium levels.

The association between blood selenium levels and NAFLD risk remains inconclusive^[43]^. Epidemiological studies in Chinese and American populations have reported a positive correlation between selenium levels and NAFLD incidence^[42,46–48]^. However, a meta-analysis showed no clear association between selenium levels and NAFLD^[34]^. Basic research studies have indicated that dietary selenium intake can modulate NAFLD progression^[49]^. In rodent models of NAFLD, an increase in blood selenium levels alleviated inflammation, lipogenesis, lipid metabolic dysfunction, and oxidative stress, thereby delaying the progression from hepatic steatosis to liver fibrosis and cirrhosis^[50,51]^. These conflicting results warrant attention. In epidemiological studies, selenium levels reflect the composite effect of baseline biological selenium and exogenous supplementation. In populations with NAFLD, baseline selenium levels may be higher due to overnutrition. Higher selenium levels might be a consequence of NAFLD rather than its cause, highlighting the need for long-term longitudinal studies to address the limitations of cross-sectional designs. Our mediation analysis revealed that blood selenium levels accounted for 13.272% of the negative mediating effect in the association between NAFLD and liver cirrhosis, suggesting that increasing blood selenium levels may slow the progression from NAFLD to cirrhosis. Further investigations are required to clarify the mechanisms through which selenium level impacts the pathogenesis and progression of chronic liver diseases.

According to subgroup analysis, patients with hypertension or females may benefit more from selenium supplementation. In hypertensive patients, increased oxidative stress may lead to a higher demand for selenium levels^[44]^. Higher selenium levels in female reproductive tissues (especially during pregnancy), which are used to regulate estrogen synthesis and maintain female reproductive capacity, may increase selenium requirements in women^[52,53]^. Moreover, sex differences in selenium levels have been widely reported in previous studies^[45,54]^, and more research is needed to explain the reasons for these sex-specific effects.

This study had certain limitations. First, this study adopted VCTE as the diagnostic criterion for liver diseases, which may have led to a certain degree of misclassification. Second, owing to limitations in data acquisition, only a limited variety of blood element levels were evaluated. Additionally, the cross-sectional nature of this study restricts its ability to make definitive causal inferences, and long-term longitudinal studies will help clarify the causal relationship. The population cohort from a single country limits extrapolation of the results, and studies across multiple countries are necessary to determine the benefit thresholds of blood selenium levels in different populations.

## Conclusion

In summary, this study elucidated the association between blood mixed metals and liver fibrosis/cirrhosis risk, revealing an L-shaped nonlinear relationship between blood selenium and liver fibrosis/cirrhosis. Blood selenium negatively mediates the association between NAFLD and liver fibrosis/cirrhosis. These findings highlight the need to better understand the potential role of selenium in chronic liver disease development.

## Data Availability

The datasets used for all analyses in this research are publicly available on the NHANES website (https://www.cdc.gov/nchs/nhanes/index.htm).

## References

[1] Chan A Kow A Hibi T Di Benedetto F Serrablo A. Liver resection in cirrhotic liver: are there any limits?. Int J Surg 2020;82s:109–14. 32652296 10.1016/j.ijsu.2020.06.050

[2] Ginès P Krag A Abraldes JG Solà E Fabrellas N Kamath PS. Liver cirrhosis. Lancet 2021;398:1359–76. 34543610 10.1016/S0140-6736(21)01374-X

[3] Ajmera V Kim BK Yang K. Liver stiffness on magnetic resonance elastography and the MEFIB index and liver-related outcomes in nonalcoholic fatty liver disease: a systematic review and meta-analysis of individual participants. Gastroenterol 2022;163:1079–1089.e1075. 10.1053/j.gastro.2022.06.073 PMC9509452 35788349

[4] Tan DJH Ng CH Lin SY. Clinical characteristics, surveillance, treatment allocation, and outcomes of non-alcoholic fatty liver disease-related hepatocellular carcinoma: a systematic review and meta-analysis. Lancet Oncol 2022;23:521–30. 35255263 10.1016/S1470-2045(22)00078-X PMC9718369

[5] Collaborators GC The global. Regional, and national burden of cirrhosis by cause in 195 countries and territories, 1990-2017: a systematic analysis for the global burden of disease study 2017. Lancet Gastroenterol Hepatol 2020;5:245–66. 31981519 10.1016/S2468-1253(19)30349-8 PMC7026710

[6] Huang DQ Terrault NA Tacke F. Global epidemiology of cirrhosis - aetiology, trends and predictions. Nat Rev Gastroenterol Hepatol 2023;20:388–98. 36977794 10.1038/s41575-023-00759-2 PMC10043867

[7] Li W Li X Su J. Associations of blood metals with liver function: analysis of NHANES from 2011 to 2018. Chemosphere 2023;317:137854. 36649900 10.1016/j.chemosphere.2023.137854

[8] Lee MR Lim YH Lee BE Hong YC. Blood mercury concentrations are associated with decline in liver function in an elderly population: a panel study. Environ Health 2017;16:17. 28257627 10.1186/s12940-017-0228-2 PMC5336614

[9] Werder EJ Beier JI Sandler DP. Blood BTEXS and heavy metal levels are associated with liver injury and systemic inflammation in Gulf states residents. Food Chem Toxicol 2020;139:111242. 32205228 10.1016/j.fct.2020.111242 PMC7368391

[10] Betanzos-Robledo L Cantoral A Peterson KE. Association between cumulative childhood blood lead exposure and hepatic steatosis in young Mexican adults. Environ Res 2021;196:110980. 33691159 10.1016/j.envres.2021.110980 PMC8119339

[11] Chen L Sun Q Peng S. Associations of blood and urinary heavy metals with rheumatoid arthritis risk among adults in NHANES, 1999-2018. Chemosphere 2022;289:133147. 34864016 10.1016/j.chemosphere.2021.133147

[12] Huang Q Wan J Nan W. Association between manganese exposure in heavy metals mixtures and the prevalence of sarcopenia in US adults from NHANES 2011–2018. J Hazard Mater 2024;464:133005. 37988867 10.1016/j.jhazmat.2023.133005

[13] Zhang L Yang F Li Y. The protection of selenium against cadmium-induced mitophagy via modulating nuclear xenobiotic receptors response and oxidative stress in the liver of rabbits. Environ Pollut 2021;285:117301. 34049137 10.1016/j.envpol.2021.117301

[14] Fan H Xiong Y Huang Y. Moderate selenium alleviates the pulmonary function impairment induced by cadmium and lead in adults: a population-based study. Sci Total Environ 2023;903:166234. 37572899 10.1016/j.scitotenv.2023.166234

[15] Loomba R Friedman SL Shulman GI. Mechanisms and disease consequences of nonalcoholic fatty liver disease. Cell 2021;184:2537–64. 33989548 10.1016/j.cell.2021.04.015 PMC12168897

[16] Kim D Cholankeril G Loomba R. Prevalence of fatty liver disease and fibrosis detected by transient elastography in adults in the United States, 2017-2018. Clin Gastroenterol Hepatol 2021;19:1499–1501.e1492. 32801011 10.1016/j.cgh.2020.08.017

[17] Kim D Konyn P Cholankeril G. Physical activity is associated with nonalcoholic fatty liver disease and significant fibrosis measured by fibroScan. Clin Gastroenterol Hepatol 2022;20:e1438–e1455. 34214678 10.1016/j.cgh.2021.06.029

[18] Siddiqui MS Vuppalanchi R Van Natta ML. Vibration-controlled transient elastography to assess fibrosis and steatosis in patients with nonalcoholic fatty liver disease. Clin Gastroenterol Hepatol 2019;17:156–163.e152. 29705261 10.1016/j.cgh.2018.04.043 PMC6203668

[19] Eddowes PJ Sasso M Allison M. Accuracy of fibroscan controlled attenuation parameter and liver stiffness measurement in assessing steatosis and fibrosis in patients with nonalcoholic fatty liver disease. Gastroenterol 2019;156:1717–30. 10.1053/j.gastro.2019.01.042 30689971

[20] Abeysekera KWM Fernandes GS Hammerton G. Prevalence of steatosis and fibrosis in young adults in the UK: a population-based study. Lancet Gastroenterol Hepatol 2020;5:295–305. 31954687 10.1016/S2468-1253(19)30419-4 PMC7026693

[21] Carrico C Gennings C Wheeler DC Factor-litvak P. Characterization of weighted quantile sum regression for highly correlated data in a risk analysis setting. J Agric Biol Environ Stat 2015;20:100–20. 30505142 10.1007/s13253-014-0180-3 PMC6261506

[22] Keil AP Buckley JP O’Brien KM. A quantile-based g-computation approach to addressing the effects of exposure mixtures. Environ Health Perspect 2020;128:47004. 32255670 10.1289/EHP5838 PMC7228100

[23] Rempelos L Wang J Barański M. Diet, but not food type, significantly affects micronutrient and toxic metal profiles in urine and/or plasma; a randomized, controlled intervention trial. Am J Clin Nutr 2022;116:1278–90. 36041176 10.1093/ajcn/nqac233 PMC9630859

[24] Bobb JF Valeri L Claus Henn B. Bayesian kernel machine regression for estimating the health effects of multi-pollutant mixtures. Biostatistics 2015;16:493–508. 25532525 10.1093/biostatistics/kxu058 PMC5963470

[25] Agha R Mathew G Rashid R. Revised strengthening the reporting of cohort, cross-sectional and case-control studies in surgery (STROCSS) guideline: an update for the age of artificial intelligence. Prem J Sci 2025;10:100081.

[26] Choi J Bae S Lim H. Mercury exposure in association with decrease of liver function in adults: a longitudinal study. J Prev Med Publ Health 2017;50:377–85. 10.3961/jpmph.17.099 PMC5717329 29207447

[27] Nasr P Ignatova S Lundberg P Kechagias S Ekstedt M. Low hepatic manganese concentrations in patients with hepatic steatosis - A cohort study of copper, iron and manganese in liver biopsies. J Trace Elem Med Biol 2021;67:126772. 34000573 10.1016/j.jtemb.2021.126772

[28] Zhao M Ge X Xu J. Association between urine metals and liver function biomarkers in Northeast China: a cross-sectional study. Ecotoxicol Environ Saf 2022;231:113163. 35030523 10.1016/j.ecoenv.2022.113163

[29] Wan H Jiang Y Yang J. Sex-specific associations of the urinary fourteen-metal mixture with NAFLD and liver fibrosis among US adults: a nationally representative study. Ecotoxicol Environ Saf 2022;248:114306. 36402077 10.1016/j.ecoenv.2022.114306

[30] Mohammed Nawi A Chin SF Jamal R. Simultaneous analysis of 25 trace elements in micro volume of human serum by inductively coupled plasma mass spectrometry (ICP-MS). Pract Lab Med 2020;18:e00142. 31720354 10.1016/j.plabm.2019.e00142 PMC6838531

[31] Shih CW Chen YJ Chen WL Inverse association between serum selenium level and severity of liver fibrosis: a cross-sectional study. Nutrients 2022;14: 3625. 36079882 10.3390/nu14173625 PMC9460482

[32] Nakahata Y Hanai T Miwa T. Effect of selenium deficiency on the development of overt hepatic encephalopathy in patients with chronic liver disease. J Clin Med 2023;12:2869. 37109203 10.3390/jcm12082869 PMC10143189

[33] Gao PT Ding GY Yang X. Invasive potential of hepatocellular carcinoma is enhanced by loss of selenium-binding protein 1 and subsequent upregulation of CXCR4. Am J Cancer Res 2018;8:1040–49. 30034941 PMC6048402

[34] Lin Y He F Lian S. Selenium status in patients with chronic liver disease: a systematic review and meta-analysis. Nutrients 2022;14:952. 35267927 10.3390/nu14050952 PMC8912406

[35] Gül-Klein S Haxhiraj D Seelig J. Serum selenium status as a diagnostic marker for the prognosis of liver transplantation. Nutrients 2021;13:619. 33672988 10.3390/nu13020619 PMC7918136

[36] Burk RF Hill KE. Regulation of selenium metabolism and transport. Annu Rev Nutr 2015;35:109–34. 25974694 10.1146/annurev-nutr-071714-034250

[37] Xu L Lu Y Wang N. The role and mechanisms of selenium supplementation on fatty liver-associated disorder. Antioxid (Basel) 2022;11:922. 10.3390/antiox11050922 PMC9137657 35624786

[38] George J. Determination of selenium during pathogenesis of hepatic fibrosis employing hydride generation and inductively coupled plasma mass spectrometry. Biol Chem 2018;399:499–509. 29408794 10.1515/hsz-2017-0260

[39] Liu Y Liu Q Ye G. Protective effects of selenium-enriched probiotics on carbon tetrachloride-induced liver fibrosis in rats. J Agric Food Chem 2015;63:242–49. 25513970 10.1021/jf5039184

[40] Zhang F Li X Wei Y. Selenium and selenoproteins in health. Biomolecules 2023;13:799. 37238669 10.3390/biom13050799 PMC10216560

[41] Naderi M Puar P Zonouzi-Marand M. A comprehensive review on the neuropathophysiology of selenium. Sci Total Environ 2021;767:144329. 33445002 10.1016/j.scitotenv.2020.144329

[42] Wang X Seo YA Park SK. Serum selenium and non-alcoholic fatty liver disease (NAFLD) in U.S. adults: National Health and Nutrition Examination Survey (NHANES) 2011–2016. Environ Res 2021;197:111190. 33872646 10.1016/j.envres.2021.111190 PMC8187321

[43] Polyzos SA Kountouras J Goulas A. Selenium and selenoprotein P in nonalcoholic fatty liver disease. Hormones (Athens) 2020;19:61–72. 31493247 10.1007/s42000-019-00127-3

[44] Rayman MP. Selenium and human health. Lancet 2012;379:1256–68. 22381456 10.1016/S0140-6736(11)61452-9

[45] Demircan K Chillon TS Bang J. Selenium, diabetes, and their intricate sex-specific relationship. Trends Endocrinol Metab 2024;35:781–92. 38599899 10.1016/j.tem.2024.03.004

[46] Liu J Tan L Liu Z. The association between non-alcoholic fatty liver disease (NAFLD) and advanced fibrosis with blood selenium level based on the NHANES 2017-2018. Ann Med 2022;54:2258–67. 10.1080/07853890.2022.2110277 PMC9455329 35975984

[47] Yang Z Yan C Liu G. Plasma selenium levels and nonalcoholic fatty liver disease in Chinese adults: a cross-sectional analysis. Sci Rep 2016;6:37288. 27853246 10.1038/srep37288 PMC5112507

[48] Guo W Weng T. Song Y Association of serum selenium with MASLD and liver fibrosis: a cross-sectional study. PLoS One 2024;19:e0314780. 39739920 10.1371/journal.pone.0314780 PMC11687858

[49] Zoidis E Seremelis I Kontopoulos N. Selenium-dependent antioxidant enzymes: actions and properties of selenoproteins. Antioxid (Basel) 2018;7:66. 10.3390/antiox7050066 PMC5981252 29758013

[50] Miyata M Matsushita K Shindo R. Selenoneine ameliorates hepatocellular injury and hepatic steatosis in a mouse model of NAFLD. Nutrients 2020;12:1898. 32604760 10.3390/nu12061898 PMC7353312

[51] Zhang W Zhang R Wang T. Selenium inhibits LPS-induced pro-inflammatory gene expression by modulating MAPK and NF-κB signaling pathways in mouse mammary epithelial cells in primary culture. Inflamm 2014;37:478–85. 10.1007/s10753-013-9761-5 24202549

[52] Qazi IH Angel C Yang H. Selenium, selenoproteins, and female reproduction: a review. Molecules 2018;23:3053. 30469536 10.3390/molecules23123053 PMC6321086

[53] Dahlen CR Reynolds LP Caton JS. Selenium supplementation and pregnancy outcomes. Front Nutr 2022;9:1011850. 36386927 10.3389/fnut.2022.1011850 PMC9659920

[54] Schomburg L Schweizer U Hierarchical regulation of selenoprotein expression and sex-specific effects of selenium. Biochim Biophys Acta (BBA) - Gen Subj 2009;1790:1453–62. 10.1016/j.bbagen.2009.03.015 19328222

